# Key stakeholders’ experiences, knowledge and perspectives regarding care quality for breast cancer in South-West Nigeria

**DOI:** 10.3389/fonc.2025.1418649

**Published:** 2025-05-02

**Authors:** Adewumi Alabi, Victoria Ainsworth, Abdulrazzaq Lawal, Darya Kizub, Jennifer Chin, Carly Woodmark, Olubukola Omidiji, Bolanle Adegboyega, Anthonia Sowunmi, Adedoyin Ogunyemi, William Swanson, Adedayo Joseph, Wilfred Ngwa

**Affiliations:** ^1^ Department of Radiation Biology, Radiodiagnosis and Radiotherapy, College of Medicine, University of Lagos, Lagos, Nigeria; ^2^ National Sovereign Investment Agency-Lagos University Teaching Hospital (NSIA-LUTH) Cancer Centre, Lagos University Teaching Hospital, Lagos, Nigeria; ^3^ Department of Radiation Oncology and Molecular Radiation Sciences, Johns Hopkins University, Baltimore, MD, United States; ^4^ Department of Physics, Medical Physics, University of Massachusetts Lowell, Lowell, MA, United States; ^5^ Department of Surgery, College of Medicine, University of Lagos, Lagos, Nigeria; ^6^ Department of Breast Medical Oncology, Division of Cancer Medicine, MD Anderson Cancer Center, Houston, TX, United States; ^7^ Project Management, Fred Hutch Cancer Centre, Seattle, WA, United States; ^8^ Department of Community Medicine, College of Medicine, University of Lagos, Lagos, Nigeria; ^9^ Department of Radiation Oncology, Weill Cornell Medicine, New York, NY, United States

**Keywords:** breast cancer, patient experiences, continuing medical education (CME), delay of care, Nigeria, global health

## Abstract

The landscape of breast cancer care in Nigeria is complex, with various structural and individual barriers impacting patient care. Breast cancer (BC) is the most common cancer and a leading cause of cancer deaths among women worldwide. In Africa, the cancer burden is expected to rise significantly, with projections estimating an increase of 50% by 2050. Rising incidence rates and barriers to care contribute to a healthcare crisis, leading to late-stage presentation and high mortality rates for women with breast cancer in Nigeria. Quality healthcare must be patient-centered, involving stakeholders - patients, clinical and community partners, and other healthcare stakeholders to achieve a desired outcome. Understanding the cancer journey from different perspectives allows for targeted approaches for increasing access to quality healthcare as well as reducing morbidity and mortality rates. To address this, healthcare provider perspectives about breast cancer care were compared with the lived experiences of breast cancer patients to emphasize the need to increase access and quality of care. A mixed method study was conducted in 2 phases: Phase I: 3 Focus group discussions (FGDs) with breast cancer patients and their care givers were conducted at the NSIA-LUTH Cancer Centre in Lagos, Nigeria. Phase II: A pre and post-survey of a continuing medical education course focused on breast cancer was delivered to healthcare providers in southwest Nigeria. Survey responses regarding causes for delays and barriers to care indicated financial strain, fear, and alternative treatments as the largest hurdles, coinciding with patient testimony from the FGD. Fear of mastectomy was a perceived barrier to care for 90% of healthcare providers while 87% and 86% of providers perceived seeking spiritual and herbal treatments as the largest delays of care. Despite this, a significant number of focus group participants (39%) presented within the first month of noticing a breast symptom to a proper healthcare provider. Data from our study reports that 70% of patients receive help from family to fund treatment highlighting why cancer can be a poverty trap for families and the need for universal health insurance. Half of the focus group participants had a positive interaction with their doctors, with the rest reporting neutral (19%) or even negative (31%) interactions. Our study also reports 42% of healthcare providers feeling only “somewhat” qualified to deal with breast cancer, highlighting the significant need for more education, with a further 14% feeling neutral or negative about their qualification, a potential contributing factor in negative interactions recalled by patients. Knowledge increase was consistent for best practice diagnostic modalities among healthcare providers (p < 0.05). At the same time, items related to symptoms and risks of breast cancer had inconsistent knowledge increases, indicating why further courses like these should be pursued. With the success of the course and the inspiration of breast cancer survivors, a proposed expansion into community awareness is discussed along with enlisting local practitioners in the fight against breast cancer in hopes of lowering the barriers to and delays of care in Nigeria.

## 1 Introduction

The landscape of breast cancer (BC) care in Nigeria is complex, with various structural and individual barriers impacting patient care. Worldwide, Breast cancer is the most common cancer and the most frequent cause of cancer death among women (1). The cancer burden in Africa is expected to increase significantly. According to the 2020 GLOBOCAN data, 186,598 breast cancer cases were reported in Africa with 85,787 related deaths (2). By 2050, these numbers are projected to rise to an estimated 50% (3). Rising incidence rates, combined with barriers to care, create a healthcare crisis.

Structural barriers include provider knowledge, education and training, provider perception of breast cancer, center location, and availability of infrastructure. Individual barriers include a lack of patient knowledge, financial constraints, and lack of an adequate support system. These barriers to care are contributing factors behind late-stage presentation and the consequent high mortality rates of women with breast cancer in Nigeria (4–6). While reported cancer incidence rates in low and middle-income countries (LMICs) are lower than those of high-income countries (HICs), overall mortality rates are much higher in countries in Sub-Saharan Africa owing to inadequate screening and detection methods, as well as limited treatment and palliative care options (7).

Quality healthcare is a goal that every practice, hospital, and institution aims for whether it is in everyday health maintenance, or the difficulties of cancer care. This healthcare must be patient-centered involving stakeholder involvement - patients, clinical and community partners, and other healthcare stakeholders to achieve a desired outcome (8). “Stakeholder” in this context is an individual or group who is responsible for or affected by health- and healthcare-related decisions (8). This individual or group play specific roles that contribute immensely to ensuring quality care which will impact patient survival. Understanding the cancer journey from different perspectives allows for targeted approaches for increasing access to quality healthcare as well as reducing morbidity and mortality rates. The identified deficiencies served as a background to develop a CME course to better equip providers to address these issues when caring for breast cancer patients. These comparisons highlight the interconnectedness of barriers to care and provider knowledge weakness that in turn can inform potential curricula for continuing medical education (CME) courses.

Our study employs a mixed-method design to examine healthcare providers’ (HCP) perspectives on breast cancer care. It will also compare these insights with the lived experiences of breast cancer patients and their caregivers. The goal is to provide continuing medical education (CME) to health care professionals by key stakeholders including breast surgeons, breast radiologists and clinical oncologists, while also providing recommendations and strategies that will help reduce barriers associated with breast cancer treatment to relevant authorities.

## 2 Materials and methods

### 2.1 Study design

A mixed-method study design was used. The study was in 2 phases, this approach was adopted to aid in the collection of different but complimentary data at the study sites to enrich the interpretation of the results.

### 2.2 Study location

This study was conducted in the southwestern part of Nigeria (Lagos State). Although ideally assumed to be a Yoruba community, the ethnic diversity in the region is rich and well-distributed. English Language is an official language in Nigeria, and researchers confirmed to see if all participants understood the language. Lagos State is the commercial center of Nigeria. The focus group discussion was conducted at the NSIA-Lagos University Teaching Hospital (LUTH) Cancer Centre (NLCC), Lagos a foremost cancer center in the region that receives referrals from within and outside of the region. The center is a state-of-the-art Oncology center equipped with modern infrastructures including the 3 latest Varian LINAC machines for radiotherapy services. The chemotherapy clinic runs 5days a week within the NLCC-LUTH cancer center of the Lagos University Teaching Hospital with an average of 10 breast cancer patients receiving chemotherapy treatment per clinic. The hospital also has a One Stop Breast Clinic, a multidisciplinary clinic where all breast services are offered every Wednesday with an average of 10 new cases per clinic. The Continuing Medical Education was done at the quarterly meeting of the Association of General and Private Medical Practitioners of Nigeria (AGPMPN) in Lagos. Ethical approval was obtained from institutional HREC.

### 2.3 Study population

#### 2.3.1 Phase I

##### 2.3.1.1 Patient focus group

Sampling method used was a purposive sampling technique. All consenting new breast cancer patients presenting for the first time to the clinic on the day of the focus group discussion were included in the study. The FGD patients were recruited one day in each month (August, September and November 2022) at the NSIA-LUTH Cancer Centre.

Three focus groups had a total of 23 participants with 9, 8 and 6 in each group respectively of women who consented on the allocated date. Study participants filled out demographic information including name, age, gender, family type, occupation, level of education, average monthly income, and marital status. Interviews were modeled around qualitative methods, which were able to capture information that contextualized answers to interview questions. Psychological and socioeconomic factors were investigated during the FGDs, including emotional, financial, and physical well-being within and outside of the clinical setting as it pertained to barriers faced during their breast cancer journeys.

The in-depth interviews (IDIs) were conducted face-to-face using an IDI guide to interview a total of twenty three (23) breast cancer patients in 3 FGDs. The interviews were conducted in the research room of the cancer center within the hospital, which was private. The interviews lasted an average of 45–60 minutes and were recorded on audiotapes. Before the interviews, the researcher explained the objectives of the study, assured the participants of confidentiality, and obtained permission to use a digital voice recorder which was later transcribed. The interviews were facilitated by a moderator and a note taker.

Questions asked related to the timeline between symptom onset and presentation to a proper HCP, satisfaction with provider response, and problems or issues faced in pursuing diagnosis and treatment. Interview items also included questions on who made up their support system (i.e. who did they first tell)? and funding questions (i.e. how did you finance your treatment)?.

##### 2.3.1.2 Data analysis

Each interview recording was transcribed verbatim into Word documents by 2 researchers J.C. and V.A. to identify patterns and themes common across participants’ experiences. The researchers read the transcripts to develop the coding guide, which aligned with the questions/sections in the quantitative instrument, and all the researchers discussed the data to ensure correctness. NVIVO version 12 was used to analyze the data.

#### 2.3.2 Phase II

##### 2.3.2.1 Methods for continuing medical education course on breast cancer

A one-day CME for HCPs was held jointly with the Association of General and Private Medical Practitioners of Nigeria (AGPMPN) in Lagos during one of their quarterly meetings. This study involved a purposeful sample of key stakeholders, comprising 71 medical doctors with experience in providing primary care for breast cancer patients. Topics for the in-person CME course titled “Advances in Multidisciplinary Management of Breast Cancer” included breast cancer risks and symptoms, diagnostic methods including various imaging modalities and testing options, breast cancer facts and treatment modalities. The CME presentation content was taken by the Breast radiologist, Breast surgeon and Clinical oncologists This course was advertised via social media platforms and posters made by the sponsoring associations. Advertisements targeted health care professionals within the AGPMPN who form the first point of contact for patients before referral to specialists. Questionnaires to assess HCP knowledge before and after the course were composed in-house using various publications concerning breast cancer diagnosis, treatment and delay of care as seen across the African continent, in Sub-Saharan Africa, and in Nigeria specifically with contextualization of the patient experience informed by FGDs as they related to finances, awareness and emotional responses with providers (4, 9–13). Key stakeholders including breast radiologists, breast surgeons, and clinical oncologists gave 45mins lecture on cancer care and answered relevant questions asked by participants. Additionally, a post course evaluation was collected.

##### 2.3.2.2 Data collection and analysis

The pre- and post-course self-administered questionnaire collected demographic data, provider knowledge and comfort with diagnosing and treating patients with breast cancer, where they go for healthcare, perceived barriers to care and additional items related to their practice. The patterns and themes identified during the FGDs with patients were then compared with the HCP assumptions on barriers to care and comfort level when dealing with breast cancer.

Questions pertaining to specific diagnostic tests, treatment modalities, and various facts about breast cancer and its symptoms, causes, and risks were asked in both pre- and post-course evaluations. While most questions asked were true/false or yes/no/don’t know, some included short-answer responses related to symptoms and referral activity. Answers from both evaluations were tabulated in excel and sent to a secondary researcher to analyze. The quantitative data were entered into the Statistical Package for the Social Sciences (SPSS version 21) statistical software. Statistical significance of nominal data was assessed using Pearson’s Chi Square test with p value threshold of <0.05.

## 3 Results

### 3.1 Focal group discussions - patient perspective

#### 3.1.1 Socio - demographics

The three focus groups had up to six participants each, ranging in age from 26 to 76 years for a total of 23 female participants with 9, 8 and 6 individuals in each group respectively. Majority of participants were in the age groups 41–50 and 51–60 years, with 9 (39%) and 6 (26%) respectively. Of these participants, 30.4% (n=7) worked in business or trade,74% (n=17) were married, 52% (n=12) had tertiary education, and 30% (n=7) had a monthly income between ₦50,000 ($30) and ₦100,000($60) {*Xe.Com converter accessed 04/11/2024*} while 26% (n=6) had no livable income (**Table 1**).

**Table 1:** Demographic information for focus group participants.

Sex	Total	Percent
Male	0	0
Female	23	100%

Age	Total	Percent
21-30	1	4%
31-40	2	9%
41-50	9	39%
51-60	6	26%
61-70	4	17%
71-80	1	4%

Marital Status	Total	Percent
Single	2	9%
Married	17	74%
Widowed	3	13%
Divorced	1	4%

Education	Total	Percent
Primary	2	9%
Secondary	6	26%
Tertiary	12	52%
Post-Tertiary	3	13%

Monthly Income	Total	Percent
₦20000-₦50000	4	17%
₦50000-₦100000	7	30%
₦100000-₦150000	3	13%
₦150000-₦200000	1	4%
₦200000-₦250000	1	4%
₦200000-₦300000	1	4%
Nil	6	26%

Occupation	Total	Percent
Academic (Current or Retired)	3	13.0%
Business and Trade	7	30.4%
Catering	1	4.3%
Civil Service (Current or Retired)	6	26.1%
Entrepreneurship	1	4.3%
Fashion	1	4.3%
Hair Dressing	2	8.7%
Homemaking	1	4.3%
Student	1	4.3%

n% < 100% due to rounding.

#### 3.1.2 Symptoms and presentations

**Figure 1:**
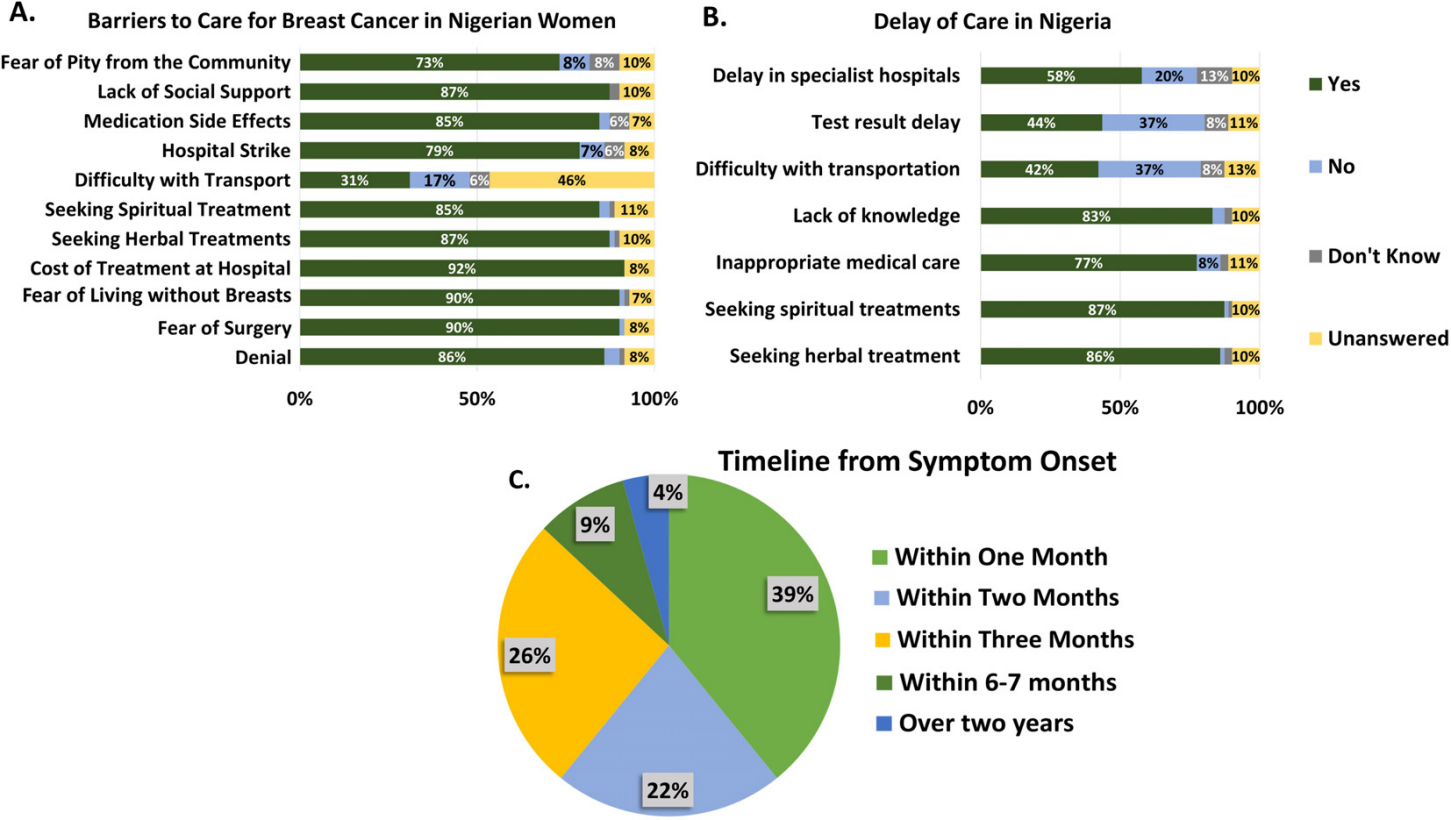
Provider responses about barriers to care **(A)** and delays of care **(B)** in Nigeria contrasted with the reported patient timeline **(C)** between symptom onset and seeing a proper healthcare professional.

Thirty-nine percent of patients (n=9) saw their HCP within one month of noticing symptoms and commenced care shortly after (**Figure 1**). Twenty-six percent and 22% of patients saw their healthcare provider within 2 and 3 months, respectively, while 4% saw a healthcare provider after 2 years. Those that had longer timelines discussed not recognizing their symptoms as breast cancer, pursuing local treatment providers, getting misdiagnosed and prescribed inappropriate treatment as the reasons for the longer timelines. In one case, the lack of appropriate diagnosis from an HCP resulted in a delay close to four years before getting accurately diagnosed and starting treatment.

**Figure 2:**
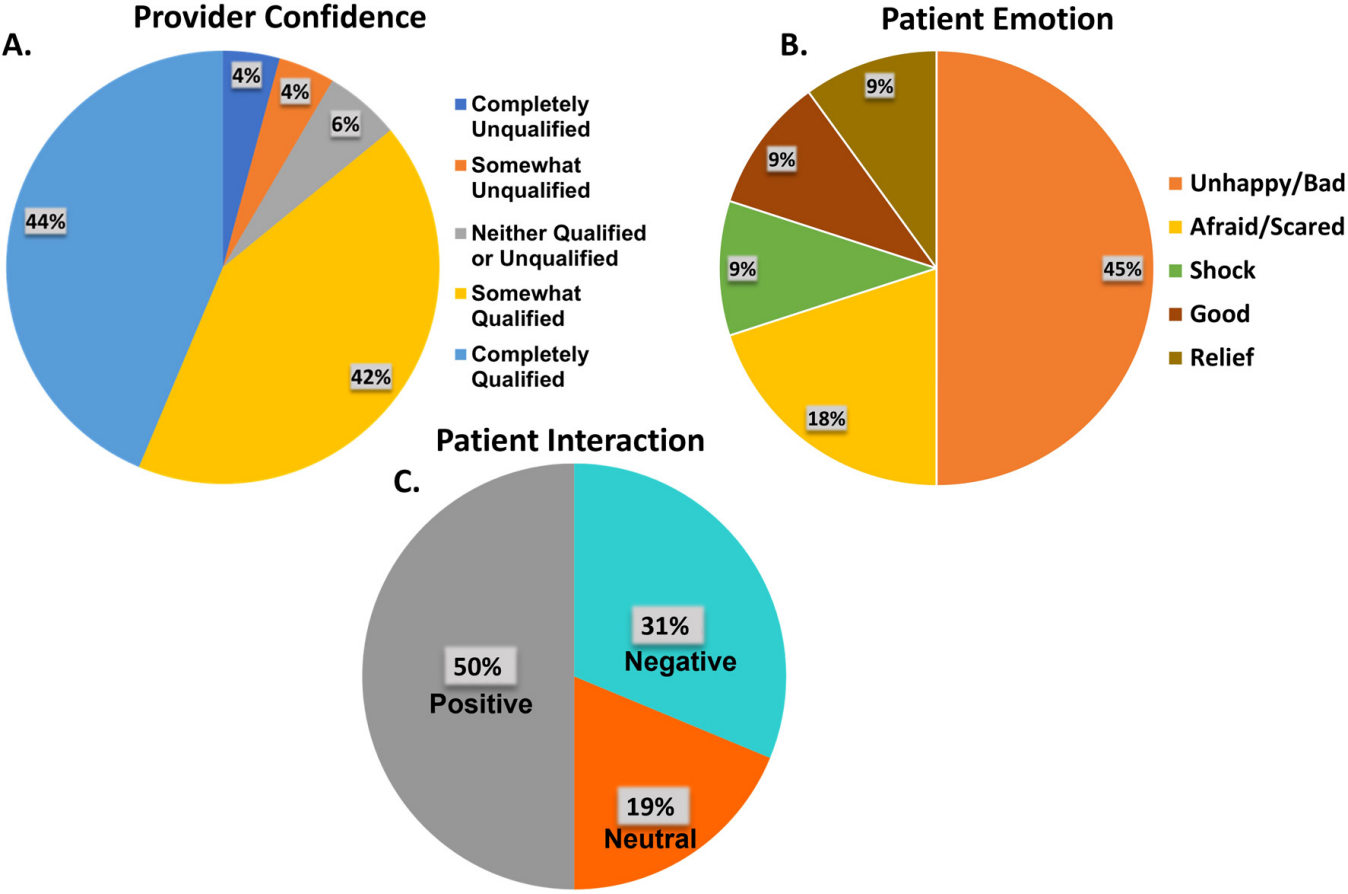
**(A)** Health care provider self-evaluated confidence with treating breast cancer. **(B)** Patient emotional state when diagnosed. **(C)** Patient-reported evaluation of interaction with their provider.

Participants in the focus groups frequently expressed their fear of death related to a breast cancer diagnosis, as well as the fear of abandonment by family or the community. Emotional responses varied among them: 45% reported feeling unhappy or sad, 18% felt afraid or scared, and 9% expressed feelings of shock, relief, or even a sense of being good. Many patients remarked that cancer felt like a death sentence. One patient even recalled being so frightened that she remained silent for several months (**Figure 2**).

#### 3.1.3 Interaction with providers

Of the sixteen FGD participants who discussed how they felt with their providers, 50% (n=8) reported positive interactions with 19% (n=3) and 31% (n=5) reporting neutral and negative interactions respectively (**Figure 2**). One patient recalled getting so frustrated with the number of tests and lack of explanation from her doctor that she stopped attending the clinic, further delaying her diagnosis before finally returning to a different provider. The contrast between the impact had by positive and negative interactions can be seen explicitly from the patient’ s testimonies. Patient A had a positive interaction, recalling “*The doctor shout. Madame, this is cancer … He said Madame I am telling you this is cancer. It is not killing people at least they need to remove my breast I can still survive (sic). This is what the doctor told me.”* Patient B, however, had a negative interaction, recalling*: “I left because the doctor did not give me hope for living. You know, when you have cancer, it is like a death sentence. When I heard it, I was so scared. For one month I did not* sp*eak. In the night I would just sit down, and I would be crying*.”

Patient C recalled: “…*I gave [the doctor] the initial report. He said he would look for a second opinion. I went and he was like no this cannot be for you. He said to please do another test. It will be expensive for you to manage to do it. So, I went for the third opinion and waited for the results…*.”

#### 3.1.4 Family support

Most funding was from family members 70% (n=16), Church 30% (n=7) and Friends 22% (n=5). Additional funding sources included patient contributions at 9% (n=2), community support at 9% (n=2), and clinical trials at 4% (n=1), along with non-governmental organizations (NGOs) or combinations of these sources (**Table 2**). Despite the preponderance of family support a few patients recollected being abandoned by their family, one patient gave the context that her family thought she would die, so they abandoned her, while another patient, who had already lost her husband, was abandoned by her relatives, leaving her to provide for both her children and her treatment alone.

**Table 2:** Funding sources as reported by focus group participants.

Funding Source	n	n%
Family	16	70%
Church	7	30%
Friends	5	22%
Self	2	9%
Community	2	9%
NGO fund	2	9%
Clinical Trial	1	4%

Participants included multiple funding sources leading to > 100% sum.

Most patients confided in family members about their diagnosis first, most commonly their husbands before anyone else. This formed their primary support with one patient emphasizing the importance of having a strong support system through her diagnosis and treatment journey, as her daughter recalled “We (*Her children) found out first before [her]. We were allowed to talk to ourselves and embrace before we broke the news to her. She received it better. [She was] the one encouraging us. We have been together as a team*.”

#### 3.1.5 Barriers

Some patients emphasized the impact hospital strikes had on their ability to start treatment. Finance was another major barrier, As patient D recalled, “*I had 50,000 Naira ($50), when I found out. I was crying because I had no money to start the treatment. But fortunately, I told a family member, and he gave me some money, so I started the treatment*…” Patient D also sought means to support herself, recalling that “*Fortunately I applied for a job they called me to come work. I said to them, I would rather make this money and keep for my children. If I survive [the cancer]…*”.

### 3.2 Providers perspective

#### 3.2.1 Pre-course survey

##### 3.2.1.1 Provider sociodemographic data

Seventy-one HCPs participated in the continuing medical education course ranging from 18 to 65+ years. Males constituted 62% (n=62), while females represented 13% (n=9). The predominant age group was 54% (n=66), aged 56–65 years. Additionally, the majority were of the Yoruba ethnicity at 47% (n=66). They included a variety of occupations within the healthcare field with a wide range of experience (70% had practiced for over 10years). Occupations included general medical doctors 45% (n=32), while others were primary care doctors at both public and private practices, family or pediatric doctors, nurses, residents, and medical students.

When asked about their own health care, providers indicated that although they primarily bring themselves and their families to larger hospitals and clinics, many still seek alternative treatments to supplement their care. Provider demographic data is presented in **Table 3**. The sociodemographic and personal healthcare questions were only present in the pre-course survey. The full questionnaire can be found in the associated **Supplementary Material**.

**Table 3:** Demographic data reflecting the attendance of the continuing medical education course on breast cancer.

Sex	n	n%
Male	62	87%
Female	9	13%

Age		
18-25	6	8%
26-35	8	11%
36-45	3	4%
46-55	7	10%
56-65	38	54%
65+	9	13%

Ethnic Background		
Yoruba	47	66%
Ibo	14	20%
Edo	1	1%
Ogu	1	1%
Urhobo	3	4%
Afemai	2	3%
Efik	3	4%
Other	1	1%

Education	Total	Percent
Secondary Schooling	1	1%
Some Tertiary	6	8%
Tertiary	14	20%
Post-Tertiary	50	70%

Occupation		
Medical Student	5	7%
Resident Doctor	6	8%
Nurse	2	3%
General Medical Doctor	32	45%
Family Doctor/Pediatrician	2	3%
Private Practice Doctor	12	17%
Medical Officer	2	3%
Misc. Undisclosed Position	10	14%

Years in Healthcare		
<1 year or Undisclosed	5	7%
1-5 Years	5	7%
6-10 Years	5	7%
>10 Years	56	79%

n%<100% due to rounding.

##### 3.2.1.2 Knowledge regarding signs and symptoms

Provider comfort with treating breast problems was self-assessed before the course on a scale from completely unqualified (4%, n=3) to completely qualified (44%, n=31), 42% felt somewhat qualified (**Figure 2**). Most providers (42%, n=30) reported seeing between one and five patients with breast complaints in the past six months. Seventeen percent (n=12) attended to six to ten patients, while 15 providers did not see any women with breast complaints during this time (**Table 4**). The array of breast problems presented at their clinics included benign breast lumps (45%, n=32), breast abscess (21%, n=15), mastitis (17%, n=12), galactorrhea (11% n=8) and fibrocystic breast disease (8%, n= 6). Other mentioned conditions included pain, breast ulcers, hotness, fungal infections, retracted nipples, skin sinuses, lipomas, boils, nipple discharge, swollen breasts, and puerperal mastitis (**Table 5**).

**Table 4:** Average number of patients presenting with a breast problem over the past six months to providers taking the CME.

Number of Women Presenting with a Breast Problem in the 6 Months Prior to CME	n	n%
Don’t know/Unanswered	3	4%
0	15	21%
1-5	30	42%
6-10	12	17%
10-20	3	4%
>20	8	11%

**Table 5:** Typical breast problems seen by healthcare providers participating in the CME within the past 6 months.

Breast Problems	n	n%
Benign Breast Lump/Fibroadenoma	32	45%
Breast Abscess	15	21%
Mastalgia/Mastitis	12	17%
Galactorrhea	8	11%
Fibroadenosis/Fibrocystic breast disease	6	8%
Nipple Discharge	4	6%
Pain	3	4%
Swollen Breast	3	4%
Boil	2	3%
Breast Cysts	2	3%
Lactational Mastitis	2	3%
Breast Hotness	1	1%
Breast Ulcer	1	1%
Fungal Infection Under Breast	1	1%
Lipoma	1	1%
Puerperal Mastitis	1	1%
Retracted Nipple	1	1%
Skin Sinus	1	1%
Others (Dysmenorrhea, Abdominal swelling, Post coital bleeding)	3	4%

Participants allowed to have multiple answers.

##### 3.2.1.3 Knowledge regarding imaging

Providers’ understanding of the typical diagnostic tests used for breast problems to detect potential breast cancer reveals patients presenting with breast symptoms primarily involve referrals for mammography (73%, n=52). Additional tests include CT imaging (46%, n=33), biopsy/histology (45%, n=32), and ultrasound scans (34%, n=24). Other diagnostic tools include CT scans, biopsies, and ultrasounds (**Table 6**).

**Table 6:** Pre-CME: Typical diagnostic tests given for breast problems to detect breast cancer.

Test	n	n%
Mammogram	52	73%
CT Imaging	33	46%
Biopsy/Histology	32	45%
Ultrasound Scan	24	34%
Bloodwork	19	27%
Cytology	17	24%
Physical Exam	9	13%
Others	7	10%

Participants allowed to choose multiple answers.

#### 3.2.2 Post-course survey

##### 3.2.2.1 Knowledge regarding breast cancer symptoms, causes and risk factors

**Figure 3:**
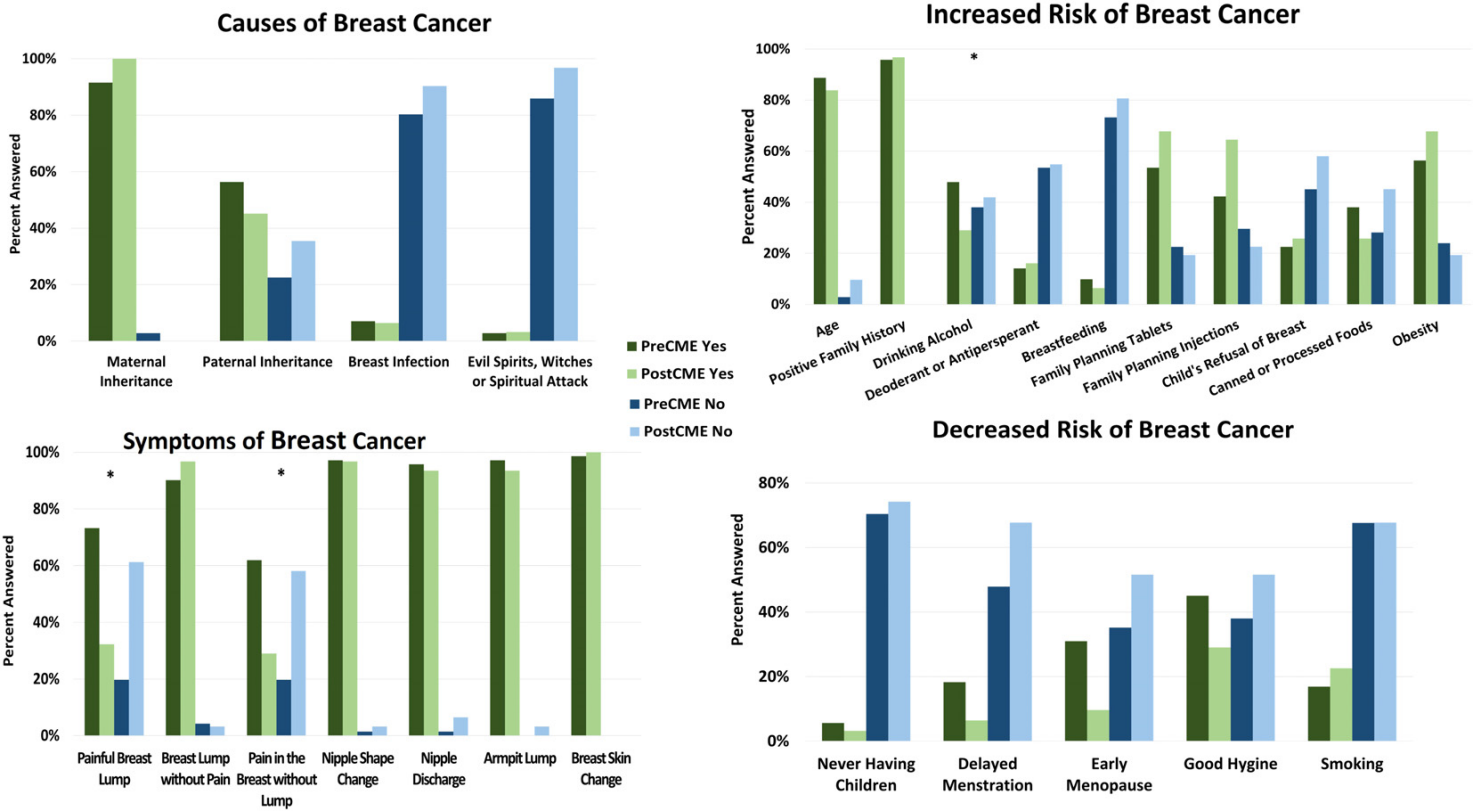
Provider knowledge increase pre- and post- CME course on breast cancer. Starting from top left going clockwise, the graphs show causes of breast cancer, causes of increased risk, causes of decreased risk and symptoms of breast cancer. Green represents “yes” while blue indicates “no”. Dark colors indicate pre-CME answers while lighter colors indicate post-CME answers. Asterisks indicate statistical significant change in answers.

Thirty-one HCPs participated in the post-course survey. Providers were asked to indicate common symptoms of breast cancer along with causes and risk factors (**Figure 3** **).** Most symptoms of breast cancer were correctly identified both pre- and post- course except painful breast lump and breast pain without lump both of which increased from 20% answering correctly to 61% and 58% respectively, both of whose responses indicated statistically significant knowledge increase (p= 0.00045 and 0.0012) (**Figure 3**).

Many providers were aware that breast cancer could be inherited from both maternal and paternal lines, both before and after CME. There is however a slight decrease in correct answers relating to paternal inheritance, but this change is slight and not deemed statistically significant (p=0.58). Other causes of breast cancer, including breast infection and beliefs in ‘evil spirits, witches, and spiritual attacks,’ clearly show a trend where participants decisively responded “no” to these.

Regarding increased breast cancer risk, most items showed either stagnating correct answers or slight increase in correct answers. There was a surprising decrease in correct answers related to age and alcohol use after the post-CME session, however, the change related to age was deemed insignificant. Alcohol use, however, did show statistical significance of knowledge decrease (p=0.022). In the opposite question of factors that decrease the risk of breast cancer, all items showed some knowledge improvement, but none were deemed statistically significant.

##### 3.2.2.2 Knowledge regarding imaging and treatment

**Figure 4:**
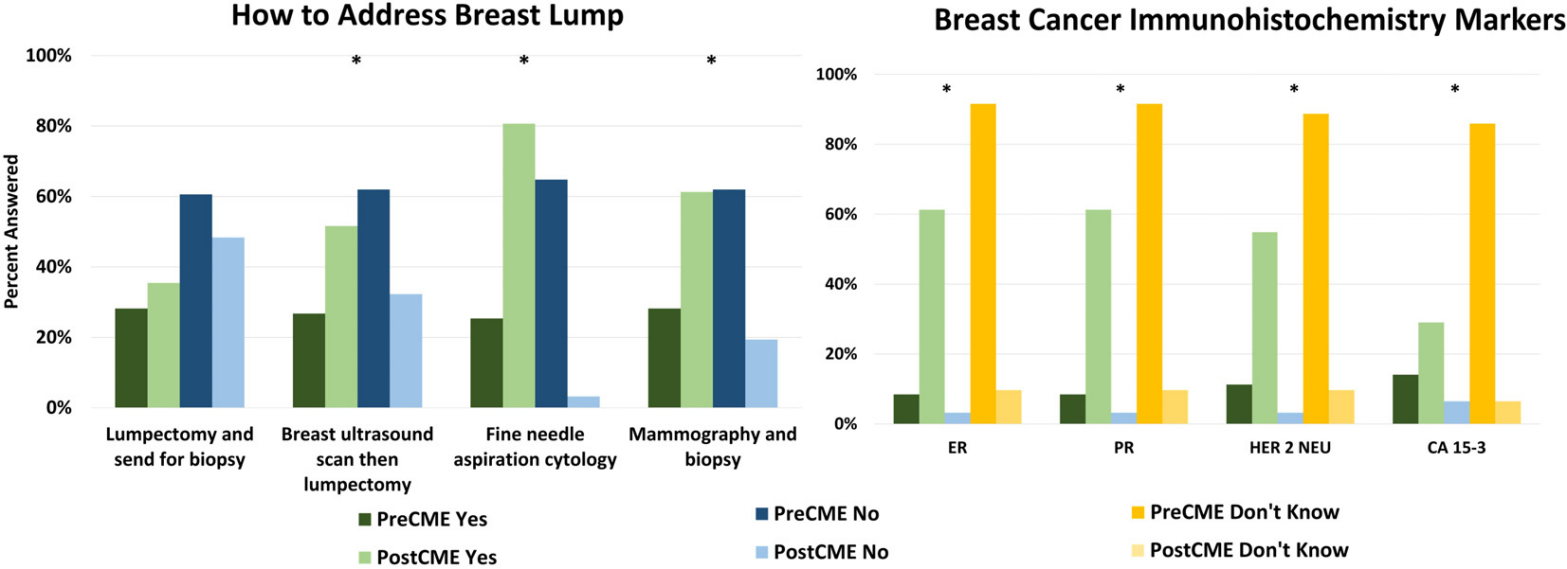
Knowledge change in providers pre- and post- CME on breast cancer. How to address a breast lump (left) and what immunohistochemistry markers to order for breast cancer. Green indicates “yes”, blue indicates “no” and yellow indicates “don’t know” with darker colors indicating pre-CME answers and lighter colors indicating post-CME answers. Asterisks indicate statistically significant change in answers.

Providers indicated a statistically significant change in how they would approach a patient presenting with a breast lump, indicating an increased use of diagnostic imaging modality options including mammography with subsequent biopsy, fine needle aspiration cytology (FNAC), and breast ultrasound followed by lumpectomy. These values were evaluated via the chi squares method and resulted in p values of 0.0013, 1.87…E-07 and 0.034 respectively, indicating both heightened awareness of these modalities and willingness to use them. Providers were split on whether or not they would order a lumpectomy with subsequent biopsy both before and after the CME, providing no significant change (p>0.05). (**Figure 4**) With chi square p values all significantly less than the threshold of 0.05, knowledge of what immunohistochemistry markers to check for had the largest increase in knowledge across providers. Before the course, most providers did not know what markers would be able to indicate breast cancer, and while after the course some participants did not answer, there was a significant increase in knowing these markers could be used. This change is displayed in **Figure 4**, with all immunohistochemistry markers asterisked to indicate statistical significance with p values of 1.9E-14, 1.9E-14, 1.5E-13, 3.4E-15 respectively. When asked about various factoids related to cancer, participants showed some improvement. When asked about mammography being able to detect breast cancer before a lump can be felt (fact 7), providers showed improvement, going from 69% answering correctly to 81%. Along with this, knowledge of treating metastasis (fact 11) went from 31% correctly answering to 48%, and mortality of breast cancer (fact 12) increased from 59% answering correctly to 65% (**Table 7**).

**Table 7:** Common facts and misconceptions about breast cancer with True/False responses as answered before (left) and after (right) CME course on breast cancer.

Both	Pre		Post	
	T	F	T	F
A painless lump is never breast cancer.	3%	**90%**	13%	**87%**
A benign lump becomes painful when it turns malignant.	31%	**54%**	32%	**65%**
Breast cancer is the most common cancer in women.	**87%**	7%	**87%**	13%
A woman less than 40 cannot develop breast cancer.	8%	**86%**	16%	**84%**
Tissue sample (histology) is the way to diagnose breast cancer.	**89%**	0%	**84%**	10%
Taking a tissue sample for histology or doing surgery causes the cancer to spread throughout the body.	10%	**69%**	26%	**68%**
Mammography can find breast cancer before a lump is felt.	**69%**	10%	**81%**	10%
Chemotherapy worsens outcomes in breast cancer	1%	**87%**	3%	**94%**
Radiation treatment worsens outcome in breast cancer	0%	**89%**	0%	**97%**
There are different types of breast cancer	**85%**	4%	**74%**	13%
If breast cancer has spread to other areas of the body it can still be eliminated from all parts of the body with medical treatment	**31%**	52%	**48%**	42%
Breast cancer is always deadly	32%	**59%**	29%	**65%**

Correct answers in bold.

[Totals between each true and false do not add to 100% due to answers of “do not know” being removed for brevity].

##### 3.2.2.3 Factors responsible for barriers and delays in care

HCP-reported opinions on barriers and delays to care are shown in **Figure 1**. Several barriers were identified and grouped into two categories: individual (or patient-related) barriers and structural barriers. Individual barriers include fear of surgery (90%), denial (86%), fear of pity from the community (73%), lack of social support (87%), medication side effects (85%), difficulty with transportation (31%), and the cost of treatment (92%) which was the largest barrier. An example of a structural barrier is hospital strikes (79%). Factors responsible for delays included seeking spiritual (87%) and herbal treatments (86%), inappropriate medical care (77%), and lack of knowledge (83%). Other factors were delays at the specialist hospital, test result delays, difficulty with transportation, and lack of knowledge.

##### 3.2.2.4 Participation in future CMEs

**Figure 5:**
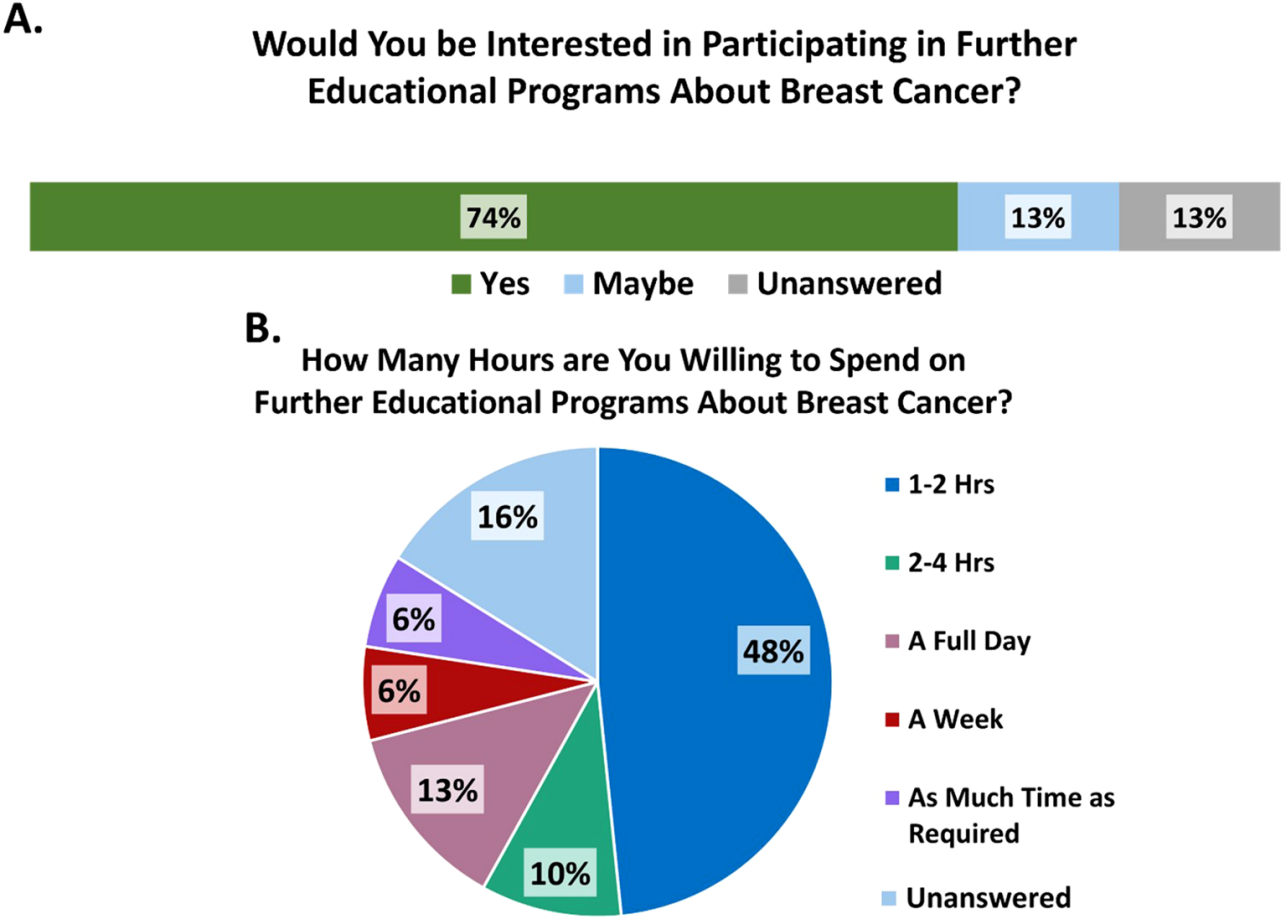
**(A)** Provider interest in participating in further educational programs on breast cancer. **(B)** Number of hours providers are willing to spend on further educational programs on Breast Cancer.

A significant 74% of participants showed interest in additional educational programs. Among them, 16% were willing to dedicate 1 to 2 hours, while 6% preferred a week-long experience. Notably, 16% chose not to respond (**Figure 5**).

## 4 Discussion

Our study showed that diverse stakeholders, including patients, care givers, primary care providers (PCPs), radiologists, surgeons, and clinical oncologists, have a role in reducing the burden encountered in a breast cancer patient’s healthcare journey. Structural and individual barriers exist and play a large role but knowing what they are and how they affect the patient is imperative to addressing them. Patient experiences are best informed via FGDs focused on breast cancer diagnosis, treatment and the impact of psychological and socioeconomic factors in hopes of bringing heightened awareness of the impact on patients in LMICs (14).

Findings from our study highlight that 39% (9 patients) were mostly age 41-50years of age. The reported mean age at diagnosis of breast cancer in Nigeria was 42.7 years (SD 12.2, range 18–85 years) (15), likewise, that reported by Zaza et al. patients had a median age of 47 (IQR: 40,58) years (16). All the patients in the study were females, with 12 of them (52%) reporting a tertiary education level. Most participants were married, comprising 74% of the sample (17 individuals). A similar demographic was noted by Zaza et al, which revealed majority of participants were also female (n=349, 99.13%) (16). Among those for whom education level was known (n=182, 52%), many had attained a tertiary level of education (16). Additionally, 261 participants (74%) were married (16), suggesting a potentially higher level of social support for breast cancer patients, as corroborated by our study where funding source was mostly from family members 16 (70%). This finding aligns with reports of family support (57.6%) in another study (17). Monthly income was between N50,000 to N100,000 for 7(30%) while 6(26%) had no livable income, this is similar to that reported that the majority of BC patients in Nigeria present late and have very low income, thus leading to inability to finance treatment (16).

The experiential responses from FGDs contextualize how barriers to care can compound and impact their care while HCP assumptions and knowledge base highlight the need for robust continuing education on specialty topics. Many patients (39%) saw a healthcare professional (HCP) within one month of beginning treatment. However, a significant number experienced delays in receiving care. Many participants discussed seeing different types of providers before finally seeing a physician with the knowledge and ability to diagnose breast cancer, which in one case ultimately caused a delay of close to four years before the patient received an appropriate diagnosis and began treatment. Frustratingly, this patient indicated that at least one provider she saw before taking it into her own hands promised to refer her to the proper care and never did. These delays were attributed to various factors, including not recognizing their symptoms as breast cancer, seeking treatment from local providers, being misdiagnosed, and receiving inappropriate treatment. Delays related to patients were reported and evaluated more often than those associated with healthcare providers or the health system, as noted by Nnaji et al. (18). This is a fairly common experience, as indicated in similar studies of breast cancer among 1429 women, the median length (months) of the diagnostic journey ranged from 11.3 (5.7-21.2) in Ugandan, 8.2 (3.4-16.4) in Zambian, 6.5 (2.4-15.7) in Namibian-black to 5.6 (2.3-13.1) in Nigerian (19). Many patients seek familiarity in their providers, going first to a friend or neighbor who is a nurse or chemist, a local health center staffed by non-physicians, pharmacists or church members before seeing a qualified physician (19). Further, once seen by a qualified physician, half (50%) had positive interactions with their provider, while the rest had neutral or negative interactions with their provider.

The fear of death associated with breast cancer diagnosis as well as the fear of familial or community abandonment were commonly brought up by the focus group participants in this study. Fear-based avoidance is commonly reported in similar studies, Ajekigbe et al. reported fear of mastectomy as a contributing factor to delayed presentation of BC patients (20), coinciding with the provider perception of fear of surgery in 90% of the responders, and this is also reflected in FGD responses presented here.

In this study, the fewest number of providers considered transportation difficulties to be a barrier to care. This contrasts with other publications, where approximately 34-42% of providers identified transportation as a delay or barrier. This discrepancy may be due to differences in the geographical locations where the studies were conducted. In this study, however, transportation was not a significant concern, as all participants lived within the city.

HCPs considered seeking care from spiritual leaders (85%), herbalists and traditional healers (87%) as a reason for delay of care. In sub-Saharan Africa, 85% of the population visits traditional healers for medical services (21). A major cause of delay in diagnosis of cancer at an early stage in Africa is the fact that many patients consult traditional healers first and are often treated by them until curative treatment cannot be undertaken (22). Our study highlighted, however, the fact that even HCPs believe they will consult alternative care practitioners if they have similar symptoms. Alternative sources of healthcare like herbalism, traditional healing and treatment from spiritual leaders play a large role in community health with average citizens as well as HCPs seeking them out. A significant portion of patients in another study, accounting for 35.1%, actively seek support from spiritual leaders or spiritual centers. However, it’s noteworthy that the majority, 54.4%, choose to visit a healthcare facility as their first step. This suggests a valuable opportunity for integrating spiritual care within the healthcare system to better support patients’ holistic needs. The World Health Organization (WHO) acknowledges the important role of traditional medicine and recommends integrating traditional healers into the health care system (23). It can be argued that these sources of care represent an important aspect of overall health, especially in emotional well- being, as these can form an integral part of one’s support system. However, it is vital to stress that these should not be the only source of care patients seek out, instead utilizing these sources as adjuvant treatments coinciding with modern medicine.

The CME course on breast cancer reported a significant increase in provider knowledge regarding the roles of mammography, fine needle aspiration cytology (FNAC), and biopsy. Additionally, the use of a breast ultrasound scan prior to a lumpectomy also yielded statistically significant results. The diagnostic test options were found to have significant (p = 0.034, 1.8E-07, 0.0012) improvement, including in the identification of specific immunohistochemistry markers. When answering true/false in regard to common breast cancer facts, there were improvements in some areas, but none with significant improvement, suggesting that future iterations of similar CME courses should focus on these facts more to improve provider confidence and reliability (**Table 7**). Research shows that continuing medical education (CME) improves knowledge and skills in medical education, ultimately leading to better healthcare outcomes (24). Results were limited by small sample size in the post-survey and high baseline knowledge. As knowledge about conducting biopsies after lumpectomies increases, it may not be statistically significant, but it can provide valuable information to some participants who are not aware of it. Additionally, this knowledge can enhance the comfort level of healthcare providers when dealing with patients presenting breast cancer symptoms. In turn, this can lead to more positive interactions and greater support for patients from their providers.

Many HCPs (74%) indicated willingness to take further training focused on breast cancer, which could be organized to incorporate the suggested collaborations. The majority’s request for 1–2 hours may be insufficient, as some results indicate that the time spent may have been inadequate to convey all necessary information. Other factors mentioned by HCPs leading to delays include issues at the specialist hospital, delays in test results, transportation difficulties, and a lack of knowledge. These issues will be addressed in expanded meetings, where solutions will be proposed to improve the referral process and ensure that every patient receives timely care and necessary attention without delays. This suggestion of an expanded continuing education course could be further impactful by curating a breast cancer awareness outreach program targeted at the community members themselves. This could help increase awareness of breast cancer while also combating the cultural taboo of discussing it (11). In resource-constrained settings, early and streamlined care is crucial for optimal outcomes. By investing in these suggestions, patients will be more likely to receive timely and appropriate treatment, even with limited resources.

It has been documented that patients with positive relationships with their care team will have an optimistic outlook on their treatment and that this will lead to a higher chance of completing treatment, and thus improve survival (25–27). Negative interactions, including the one patient who stopped attending the clinic, can leave patients feeling hopeless and unattended to. In the positive interactions discussed in the FGD, patient A felt that they had a chance at survival, while in the negative interaction, patient B left with her trust in her physician broken with no hope for survival. The lack of trust in any patient-provider relationship can lead to negative outcomes strongly supported by the predictions regarding both affectionate communication and affection deprivation where affectionate communication was found to positively relate to most outcome measures and vice versa (25). Luckily in the case of patient B, she was able to pursue a second opinion, however, many others with similar experiences do not have the ability, knowledge or financial means to seek out a second opinion, resulting in much poorer outcomes, reflecting a failure of the healthcare system.

The financial burden of the journey to care further impacts the patient’s well-being. The process of diagnosis can be long, expensive, and frustrating on its own, but when presented with further expenses incurred for treatment, patients are caught in a poverty trap that only serves to further delay getting better. Patient C recalled being referred to a national hospital and meeting with an oncologist who requested a second opinion for her, however also brought up the expense this incurred. These tests, while important and relevant for diagnosing and staging patients properly, impose increasing financial burdens. These tests come even before discussion of treatment options, which many patients do not have the means to pay for. While the two abandoned women in the FGD were supported by a community that rallied to support them, many other women in the same position are not as fortunate and are often forced to forego treatment and ultimately pass away due to their cancer. The majority of funding was from family members 70% (n=16), church 30% (n=7) and friends 22% (n=5) indicating that in poor resource settings, healthcare spending is often out-of-pocket, which is associated often with a high rate of catastrophic healthcare expenditure (28). There is limited data concerning the specific costs associated with cancer care in Nigeria, which significantly impacts patients and their families (29). This lack of information presents challenges in developing effective health policies and creating local treatment guidelines. It is essential for stakeholders to actively participate in efforts to make cancer care accessible and affordable for everyone.

With success, providers should have increased competency in treating breast cancer and have an expanded “tool-box”. The enhancement of capabilities through the collaborative efforts of stakeholders is expected to significantly improve patient outcomes and provide more comprehensive care for individuals affected by breast cancer.

## 5 Conclusion

FGDs highlighted the importance of support systems in patients’ treatment journeys. Key elements of this support include encouragement from family and community, as well as positive interactions with healthcare providers. These factors contribute significantly to the overall experience and well-being of patients during their treatment. Providing educational tools like the CME or awareness course can enhance community knowledge of breast cancer and enable quicker referrals to healthcare providers.

To promote positive patient behaviors, key stakeholders need to participate actively in community outreach efforts and provide ongoing education programs, like the one outlined here. Future initiatives should emphasize discussions about breast cancer that incorporate a broader and more diverse range of participants. This approach will help identify and address both individual and structural barriers to accessing healthcare. This study revealed a huge lacuna in the referral system of breast cancer patients to tertiary hospitals.

The importance of Continuing Medical Education (CME) programs cannot be overstated when it comes to educating healthcare professionals (HCPs) and providing them with guidance on evidence-based medicine. This is particularly crucial in low-resource settings, where these practitioners play a vital role in promoting community health. However, this guidance can only be effective if patients are also informed. Therefore, collaboration among local providers, alternative treatment practitioners, and modern HCPs is recommended. Additionally, a post-survey will be conducted to assess any changes in healthcare professionals’ attitudes toward appropriate referrals and the management of breast cancer.

## Data Availability

The raw data supporting the conclusions of this article will be made available by the authors, without undue reservation.
