# Supplementary material for: Key stakeholders’ experiences, knowledge and perspectives regarding care quality for breast cancer in South-West Nigeria
Source: Front Oncol. 2025 May 2;15:1418649. doi: 10.3389/fonc.2025.1418649 (PMC12081249; doi:10.3389/fonc.2025.1418649)
Supplement: Supplementary file 1 [file DataSheet1.pdf]

Breast Cancer Awareness Questionnaire for Community Healthcare Workers:

IRB Protocol Number 13304B

Facility Name:
Unit:
Provider Initials:
DOB: (dd/mm/yyyy) _ _ / _ _ / _ _ _ _
Interview Date: (dd/mm/yyyy) _ _ / _ _ / _ _ _ _
Contact Information:
Email:
Cell Phone number

Please fill ***2*** *copies of the informed consent form. One for you to keep and one should be returned to the research staff. Thank you!*

Section 1: We are going to start with some demographic questions.

Question 1: What is your gender?
1. Male
2. Female

Question 2: What is your age?
1. 18-25
2. 26-35
3. 36-45
4. 46-55
5. 56-65
6. Above 65
7. Don't know

Question 3: What is your religion?
1. Christian: Which denomination? _____________________
2. Muslim: Which sect? ________________________
3. None
4. Other: Please specify: _______________________

Question 4: What is your ethnic background?
1. Yoruba
2. Ibo
3. Hausa
4. Don't know
5. Other: ___________________

Question 5: What is your marital status?
1. Never married
2. Married
3. Widowed
4. Divorced
5. Separated
6. Don't know

Question 6: How many children do you have?
1. I do not have children
2. 1-2 children
3. 3-4 children
4. 5-6 children
5. > 6 children

Question 7a: What is your level of education?
1. No formal education
2. Some primary school
3. Completed primary school
4. Some secondary school
5. Completed secondary school
6. Some tertiary (university or polytechnic) education
7. Completed tertiary
8. Post-tertiary degree

Question 7b: What was the degree or what were the degrees awarded?
____

Question 8: What is your current occupation?
____

Question 9: How many years have you been working as a healthcare provider?
1. Less than 1 year
2. 1-5 years
3. 6-10 years
4. > 10 years

Section 2: Next we are going to ask about any personal experiences you may have with breast cancer.

Question 10: Has anyone in your family been diagnosed with breast cancer?
1. Yes
2. No
3. Don't know
   If yes, who? ____

Question 11: Do you know anyone, outside of your family, who has been diagnosed with breast cancer?
1. Yes
2. No
3. Don't know
   If yes how do you know them? ____

Question 12: Do you personally know any survivors (more than 5 years with no recurrence) of breast cancer?
1. Yes
2. No
3. Don't know

Section 3: Now we are going to ask about your professional experience with breast problems:

For each item choose yes or no if this is one way that you have learned about breast cancer.

Question 13: Training for your degree 1. Yes 2. No

Question 14: Educational workshop 1. Yes 2. No

If yes where: ____________________________

Question 15: Poster or billboard 1. Yes 2. No

If yes where: ____________________________

Question 16: Pamphlet 1. Yes 2. No

If yes where: ____________________________

Question 17: Television 1. Yes 2. No

Question 18: Radio 1. Yes 2. No

Question 19: Newspaper 1. Yes 2. No

Question 20: Experience Treating Patients 1. Yes 2. No

Question 21: Please list any other ways or places you have learned about breast cancer:
____

Question 22: How qualified do you feel to assess female patients with breast problems?
1. Completely unqualified
2. Somewhat unqualified
3. Neither qualified or unqualified
4. Somewhat qualified
5. Completely qualified

Question 23: Please list 5 common symptoms of breast cancer:
1.
2.
3.
4.
5.

Question 24: How many women have presented to you with a breast problem in the last 6 months?
1. 0
2. 1-5
3. 5-10
4. 10-20
5. >20
6. Don't know

Question 25: Apart from breast cancer, what breast problems have women presented to you with?

____

Question 26: What type of testing did you recommend for these women? (Please list all types that were recommended)

____

Question 27: What type of treatment did you recommend? (Please select all that apply)
1. Oral herbs
2. Application of herbal cream
3. Prayers or spiritual intervention
4. Injections
   Name of injection: ______________________
5. Tablets
   Name of tablets: ________________________
6. Referral
7. Other: ________________________________

Question 28: Did you refer these women to another healthcare provider or facility?
1. Yes
2. No
   If yes please list the three most common places you referred them to:
   ____

Section 4: Now we are going to ask some questions about where you or your family goes for healthcare and the types of care you receive.

******Only if female answer questions 29 – 38******

For each item choose yes, no or don't know if this is somewhere you would go for care if you had a breast problem:

Question 29: Herbalist	1. Yes	2. No	3. Don't know
Question 30: Traditional healer	1. Yes	2. No	3. Don't know
Question 31: Chemist	1. Yes	2. No	3. Don't know
Question 32: Pharmacy Shop	1. Yes	2. No	3. Don't know
Question 33: Primary Healthcare Center	1. Yes	2. No	3. Don't know
Question 34: Private hospital	1. Yes	2. No	3. Don't know
Question 35: General hospital	1. Yes	2. No	3. Don't know
Question 36: Specialist (teaching) hospital	1. Yes	2. No	3. Don't know
Question 37: Religious leader	1. Yes	2. No	3. Don't know

Question 38: Please list any other places you would seek care for a breast problem:

____

******If male answer questions 39- 49******

For each item choose yes, no or don't know if this is somewhere you would advise your female relative, such as wife or mother, to go for care for a breast problem:

Question 39: Herbalist	1. Yes	2. No	3. Don't know
Question 40: Traditional healer	1. Yes	2. No	3. Don't know
Question 41: Chemist	1. Yes	2. No	3. Don't know
Question 42: Pharmacy Shop	1. Yes	2. No	3. Don't know
Question 43: Primary Healthcare Center	1. Yes	2. No	3. Don't know
Question 44: Private hospital	1. Yes	2. No	3. Don't know
Question 45: General hospital	1. Yes	2. No	3. Don't know
Question 46: Specialist (teaching) hospital	1. Yes	2. No	3. Don't know
Question 47: Religious leader	1. Yes	2. No	3. Don't know

Question 48: Please list any other place you would take your female relative for a breast problem:

____

Question 49: Are you aware of breast self-examination (BSE)?

1. Yes
2. No
3. Don't know

******Only if female answer questions 50 – 55******

Question 50: Do you practice breast self-examination (BSE)?

1. Yes
2. No
3. Don't know

   If no why: ________________________________

Question 51:
Answer if Yes to 50: On average how often do you practice breast self-examination (BSE)?

1. Daily
2. Weekly
3. Monthly
4. Yearly
5. Less than yearly
6. Don’t know
7. Other: ____________________

Question 52: Describe how you complete breast self-examination: (Mark if mentioned)

1. Removes clothes above the waist
2. Places hand behind head (position)
3. Visual inspection before a mirror for symmetry
4. Check for skin changes
5. System or pattern for palpating breast (spokes, wheel, grid)
6. Checks with the pads of the fingers

Question 53: Have you had a clinical breast exam in the past twelve months?

1. Yes
2. No
3. Don’t know

If no why: __________________________________

If yes why:

1. Routine examination
2. Don’t know
3. Other: _______________________________

Question 54: Have you ever had a mammogram?

1. Yes
2. No
3. Don’t know

If no why: __________________________________

If yes why:

1. Routine screening
2. Don’t know
3. Other: _______________________________

Question 55: Would you be interested in getting a mammogram for routine screening?

1. Yes
2. No
3. Don’t Know

If no why:___________________________________

Section 5: The next set of questions is about symptoms of breast cancer.

For each item 56-62 choose yes, no or don’t know if this can be a symptom of breast cancer

Question 56: Painful breast lump	1. Yes	2. No	3. Don’t know
Question 57: Breast lump without pain	1. Yes	2. No	3. Don’t know
Question 58: Pain in the breast without lump	1. Yes	2. No	3. Don’t know
Question 59: Change in shape of the nipple	1. Yes	2. No	3. Don’t know
Question 60: Nipple discharge	1. Yes	2. No	3. Don’t know
Question 61: Lump in the armpit	1. Yes	2. No	3. Don’t know
Question 62: Skin changes on the breast	1. Yes	2. No	3. Don’t know

Question 63: What signs make you suspect that a breast problem is breast cancer? (mark if mentioned)

1. Difference in size between breasts
2. Nipple retraction
3. Skin changes (peau d’orange)
4. Enlarging mass
5. Long duration of changes
6. Ulceration of the skin

Question 64: Please select true, false or don’t know: A painless lump is never breast cancer.

1. True
2. False
3. Don’t know

Question 65: Please select true, false or don’t know: A benign lump becomes painful when it turns malignant.

1. True
2. False
3. Don’t know

Section 6: The next set of questions is about causes of breast cancer.

Question 66: Please select true, false or don’t know: Breast cancer is contagious.

1. True
2. False
3. Don’t know

Question 67: Please select true, false or don’t know: Breast cancer can run in families and be inherited

a. from the mother’s side of the family.

1. True
2. False
3. Don’t know

b. from the father’s side of the family.

1. True
2. False
3. Don’t know

Question 68: Please select true, false or don’t know: Breast cancer is caused by keeping dirty items like money in the bra.

1. True
2. False
3. Don’t know

Question 69: Please select true, false or don’t know: Breast cancer is caused by an infection in the breast.
1. True
2. False
3. Don’t know

Question 70: Please select true, false or don’t know: Breast cancer is caused by evil spirits, witches or a spiritual attack.
1. True
2. False
3. Don’t know

Section 7: The next set of questions is about risk factors for breast cancer.

Question 71: Please select true, false or don’t know: Breast cancer is the most common cancer in women.
1. True
2. False
3. Don’t know

Question 72: Please select true, false or don’t know: A woman less than 40 cannot develop breast cancer.
1. True
2. False
3. Don’t know

For each item 73-82 choose yes, no or don’t know if this increases risk of breast cancer

Question 73: Increasing age	1. Yes	2. No	3. Don’t know
Question 74: Family history of breast cancer	1. Yes	2. No	3. Don’t know
Question 75: Drinking alcohol	1. Yes	2. No	3. Don’t know
Question 76: Using deodorant or antiperspirant	1. Yes	2. No	3. Don’t know
Question 77: Breastfeeding	1. Yes	2. No	3. Don’t know
Question 78: Family planning tablets	1. Yes	2. No	3. Don’t know
Question 79: Family planning injections	1. Yes	2. No	3. Don’t know
Question 80: Child refusing to suck a particular breast	1. Yes	2. No	3. Don’t know
Question 81: Eating canned or processed foods	1. Yes	2. No	3. Don’t know
Question 82: Obesity	1. Yes	2. No	3. Don’t know

For each item 83- 87 choose yes, no or don’t know if this decreases risk of breast cancer

Question 83: Never having children	1. Yes	2. No	3. Don’t know
Question 84: Starting menstruation late	1. Yes	2. No	3. Don’t know
Question 85: Undergoing menopause early	1. Yes	2. No	3. Don’t know
Question 86: Good hygiene	1. Yes	2. No	3. Don’t know
Question 87: Smoking	1. Yes	2. No	3. Don’t know

Section 8: The next set of questions is about diagnosis and treatment of breast cancer.

Question 88: Please select true, false or don’t know: Early diagnosis of breast cancer improves outcome.

1. True
2. False
3. Don’t know

For each item 89- 92 choose yes, no or don’t know if this is an effective way to detect breast problems such as breast cancer

Question 89: Breast self-examination	1. Yes	2. No	3. Don’t know
Question 90: Clinical breast examination	1. Yes	2. No	3. Don’t know
Question 91: Mammogram (xray)	1. Yes	2. No	3. Don’t know
Question 92: Breast ultrasound	1. Yes	2. No	3. Don’t know

Question 93: Please select true, false or don’t know: Tissue sample (histology) is the best way to diagnose breast cancer.

1. True
2. False
3. Don’t know

Question 94: Please select true, false or don’t know: Taking a tissue sample for histology or doing surgery causes the cancer to spread throughout the body.

1. True
2. False
3. Don’t know

Question 95: Please select true, false or don’t know: Mammogram can find breast cancer before a lump is felt.

1. True
2. False
3. Don’t know

For each item 96-100 choose yes, no or don’t know if this is an effective treatment of breast cancer

Question 96: Herbs	1. Yes	2. No	3. Don’t know
Question 97: Antibiotics	1. Yes	2. No	3. Don’t know
Question 98: Surgery	1. Yes	2. No	3. Don’t know
Question 99: Chemotherapy	1. Yes	2. No	3. Don’t know
Question 100: Radiation treatment	1. Yes	2. No	3. Don’t know

Question 101: Please select true, false or don’t know: Chemotherapy worsens outcomes in breast cancer.

1. True
2. False
3. Don’t know

Question 102: Please select true, false or don't know: Surgery worsens outcomes in breast cancer.

1. True
2. False
3. Don't know

Question 103: Please select true, false or don't know: Radiation treatment worsens outcomes in breast cancer.

1. True
2. False
3. Don't know

Question 104: Please select true, false or don't know: There are different types of breast cancers.

1. True
2. False
3. Don't know

Question 105: Please select true, false or don't know: If breast cancer has spread to other areas of the body it can still be eliminated from all parts of the body with medical treatment.

1. True
2. False
3. Don't know

Question 106: Please select true, false or don't know: Breast cancer is always deadly.

1. True
2. False
3. Don't know

Section 9: The next set of questions is about breast cancer and delays in care in Nigeria:

Many Nigerian women are diagnosed with breast cancer at a late stage. For our research we are interested in understanding why they present late.

For each item 107- 114 choose yes, no or don't know if you believe this is a reason Nigerian women present late with breast cancer:

Question 107: Seeking herbal treatments	1. Yes	2. No	3. Don't know
Question 108: Seeking spiritual treatments	1. Yes	2. No	3. Don't know
Question 109: Inappropriate medical care	1. Yes	2. No	3. Don't know
Question 110: Lack of knowledge	1. Yes	2. No	3. Don't know
Question 111: Difficulty with transport	1. Yes	2. No	3. Don't know
Question 112: Test result delay	1. Yes	2. No	3. Don't know
Question 113: Delay in specialist hospitals	1. Yes	2. No	3. Don't know

Question 114: Other reasons for late presentation: ___________________________________

Even after they are diagnosed many Nigerian women have trouble completing their breast cancer treatment.

For each item 115- 126 mark yes, no or don't know if you believe this is a barrier to medical treatment of breast cancer for Nigerian women:

Question 115: Denial (rejecting the presence of breast lump)	1. Yes	2. No	3. Don't know
Question 116: Fear of surgery	1. Yes	2. No	3. Don't know
Question 117: Fear of living without breast(s)	1. Yes	2. No	3. Don't know
Question 118: Cost of treatments at hospital	1. Yes	2. No	3. Don't know
Question 119: Seeking herbal treatments	1. Yes	2. No	3. Don't know
Question 120: Seeking spiritual treatments	1. Yes	2. No	3. Don't know
Question 121: Difficulty with transport	1. Yes	2. No	3. Don't know
Question 122: Hospital strikes	1. Yes	2. No	3. Don't know
Question 123: Side effects of medications	1. Yes	2. No	3. Don't know
Question 124: Lack of social support	1. Yes	2. No	3. Don't know
Question 125: Fear of pity from the community	1. Yes	2. No	3. Don't know

Question 126: Other reasons for not completing treatment: ___________________________

Section 9: The next set of questions is about if you are interested in an education program.

Question 127: Would you be interested in participating in an educational program about breast cancer?

1. Yes
2. No
3. Maybe

Question 128: How many hours would you be willing to spend in an educational program about breast cancer?

1. Less than 1 hour
2. 1-2 hours
3. 2-4 hours
4. A full day
5. A week
6. Other: _________________________

Question 129: Please list any suggestions you have about the type of educational program you would be interested in:
